# Diversity and Distribution Patterns in High Southern Latitude Sponges

**DOI:** 10.1371/journal.pone.0041672

**Published:** 2012-07-24

**Authors:** Rachel V. Downey, Huw J. Griffiths, Katrin Linse, Dorte Janussen

**Affiliations:** 1 British Antarctic Survey, Natural Environmental Research Council, Cambridge, United Kingdom; 2 Sektion Marine Evertebraten I, Forschungsinstitut und Naturmuseum Senckenberg, Frankfurt am Main, Germany; National Institute of Water & Atmospheric Research, New Zealand

## Abstract

Sponges play a key role in Antarctic marine benthic community structure and dynamics and are often a dominant component of many Southern Ocean benthic communities. Understanding the drivers of sponge distribution in Antarctica enables us to understand many of general benthic biodiversity patterns in the region. The sponges of the Antarctic and neighbouring oceanographic regions were assessed for species richness and biogeographic patterns using over 8,800 distribution records. Species-rich regions include the Antarctic Peninsula, South Shetland Islands, South Georgia, Eastern Weddell Sea, Kerguelen Plateau, Falkland Islands and north New Zealand. Sampling intensity varied greatly within the study area, with sampling hotspots found at the Antarctic Peninsula, South Georgia, north New Zealand and Tierra del Fuego, with limited sampling in the Bellingshausen and Amundsen seas in the Southern Ocean. In contrast to previous studies we found that eurybathy and circumpolar distributions are important but not dominant characteristics in Antarctic sponges. Overall Antarctic sponge species endemism is ∼43%, with a higher level for the class Hexactinellida (68%). Endemism levels are lower than previous estimates, but still indicate the importance of the Polar Front in isolating the Southern Ocean fauna. Nineteen distinct sponge distribution patterns were found, ranging from regional endemics to cosmopolitan species. A single, distinct Antarctic demosponge fauna is found to encompass all areas within the Polar Front, and the sub-Antarctic regions of the Kerguelen Plateau and Macquarie Island. Biogeographical analyses indicate stronger faunal links between Antarctica and South America, with little evidence of links between Antarctica and South Africa, Southern Australia or New Zealand. We conclude that the biogeographic and species distribution patterns observed are largely driven by the Antarctic Circumpolar Current and the timing of past continent connectivity.

## Introduction

Sponges play a key role in Antarctic marine benthic community structure and dynamics [1]–[3]. Sponges are often a dominant component of many Antarctic benthic communities, but in some areas they can be patchy in distribution [4]. These animals can form heterogeneous habitats supporting some of the richest benthic communities in the Antarctic, as has been found in the Weddell Sea [5], Ross Sea [6], East Antarctica [7] and the West Antarctic Peninsula [8], [9].

Previous estimates of sponge species numbers in the Southern Ocean (SO) vary between 250–530 [10]–[14]. All four classes of Porifera (sponges), Hexactinellida, Demospongiae, Homoscleromorpha and Calcarea, are represented in the SO; the first two classes (particularly the demosponges) have been found in higher diversity and abundances, whereas the latter two are comparably rarer. Potential reasons for sponge dominance in the SO include favourable nutrient and hydrochemical conditions, with silica levels being particularly high in the SO which is important in all hexactinellid and many demosponge structural development [15], as well as the abundance of coarse terrigenous material as settlement bases, regularly deposited by glaciers and icebergs [10]. Demospongiae comprise the majority of the Antarctic Porifera in terms of species numbers [2] and have been recorded from all regions of Antarctica. Glass sponges (Hexactinellida) are an ecologically important group and are more common in the SO than in any other ocean [16], [17], with a few endemic genera dominating shelf communities, most notably the Weddell and Ross seas [13]; whereas the diversity of hexactinellid sponges in is notably higher in the Antarctic deep-sea [18]. Calcareous sponges are the least abundant and least studied class of sponges in the Antarctic and, until recently, were believed to be confined to shallow waters due to the shallowing of the carbonate compensation depth (CCD) in the SO. However, representatives have been recently found at great depths in the Weddell Sea [19].

Sponges perform an important role in benthic ecosystem communities by forming high biomass [20], [6], which is an important food source for numerous organisms, such as amphipods [5], sea stars [21], and nudibranchs [22]. Coupled with their three-dimensional structures, sponges provide heterogeneous and complex habitats, nurseries and substrate for a vast array of marine organisms [11], [13], [23], [24]. The sponge body provides microhabitat for many epibiotic species [11], [25] and larger sponges provide habitat for mobile species, such as echinoderms and holothurians, often using the sponge as a raised platform for filter feeding [23], as well as playing an important role in several fish species lifecycles [26], [27]. Some species grow upon living substrata [23], as well as on the mats of siliceous spicules from dead sponges, which also provide a home for many infaunal species. Sponges are important colonisers in early, and end-members in later community stages, recovery from iceberg disturbance [28]. The evolution of sponge epifauna and their epibiotic relationships have been suggested as major explanations for the high Antarctic benthic species richness [23]. Understanding the patterns behind their distribution and diversity will play an important part in understanding the biogeography of the SO benthos. For these reasons sponges have been recognized by policy makers in the region as important indicators of Vulnerable Marine Ecosystems (VME’s) for conservation purposes [13], [29].

The history of scientific studies of sponges in the SO and Antarctica dates back almost 140 years to HMS *Challenger* (1872–1876). The earliest attempt to use the distribution data for biogeographic analyses was carried out by Burton [30] in the reports from the Discovery expedition (1925–1927). Using records for three Porifera classes, Demospongiae, Hexactinellida and Calcarea, he found no clear differences between the sponge fauna of Antarctic Peninsula and that of the Ross Sea and a high degree of affinity between Antarctica and the sub-Antarctic and southern South America. Koltun [10], [31] considered data on ∼300 species of SO demosponges and hexactinellids, from which he defined an Antarctic biogeographic unit, with possibly distinct faunas for East and West Antarctica, a closer faunistic relationship with South America and Falklands than with Australia and New Zealand, a high level of species level endemism coupled with very little genus level endemism, wide eurybathic ranges, and a circumpolar distribution of the majority of species. Sarà et al. [32] investigated the distribution of 352 species of demosponges from the SO by dividing southernmost portion of the Southern Hemisphere into continental Antarctic, non-continental Antarctic and non-Antarctic regions. They then, arbitrarily, sub-divided the Antarctic into 40° longitudinal segments. Species lists were assembled for each of these geographic entities and the faunal similarity between entities was assessed. They discovered the existence of a distinct Antarctic Faunistic Complex (AFC) which included the continental Antarctic, sub-Antarctic Islands and the Magellan region of South America [32]. Within this AFC they found a greater similarity between the Antarctic continental sponge fauna and that of the Scotia arc, a slightly weaker similarity to the Magellan region, and very little faunal similarity between the AFC and South Africa, Australia and New Zealand. Van Soest [33] examined global demosponge distributions and found that the Antarctic grouped with the sub-Antarctic and South America at genus level and for widespread species. Tabachnick [16] researched the global distribution of hexactinellids and found that Antarctica had the highest species diversity and showed relatively high similarity to other Southern Hemisphere regions, but with a different pattern to that found for the demosponges. McClintock et al. [2] concentrated on Antarctic species, largely focusing on their ecological role. They did, however, consider the wide circumpolar and bathymetric distribution patterns of demosponges, and the diversity and richness of all three major sponge classes. Pansini and Sarà [34] undertook a regional study of the Strait of Magellan and found a close affinity (14 out of 44 species in common) with the fauna of the Antarctic continent.

A recently published biogeographic work [3] highlights how little is known about the Antarctic deep-sea fauna. Janussen and Tendal [3] report on the species collected by the ANDEEP cruises which collected bathyal and abyssal material primarily from the Weddell Sea. From these collections 76 species from all three sponge classes were examined, including the first recorded calcareous sponges from the Antarctic deep-sea. They recognise a shift in taxonomic composition, from the largely endemic sponge fauna on the Antarctic shelf to a more cosmopolitan deep sea fauna [3].

Initiatives such as SCAR-MarBIN (www.scarmarbin.be) and the Census of Antarctic Marine Life (CAML, www.caml.aq) have greatly advanced sharing and knowledge of Antarctic taxon data by collating geo-referenced species information into one central point. We still know very little about the biology of the 40 degrees of longitude spanning the Amundsen Sea, shelf underneath ice-shelves and most of the continental slope and deep sea. Here we present the analyses of most comprehensive biodiversity and biogeography dataset available to date for SO and Antarctic sponges that we compiled and have made publically available through SCAR-MarBin and CAML.

## Results

### Species Records and Richness

**Figure 1:**
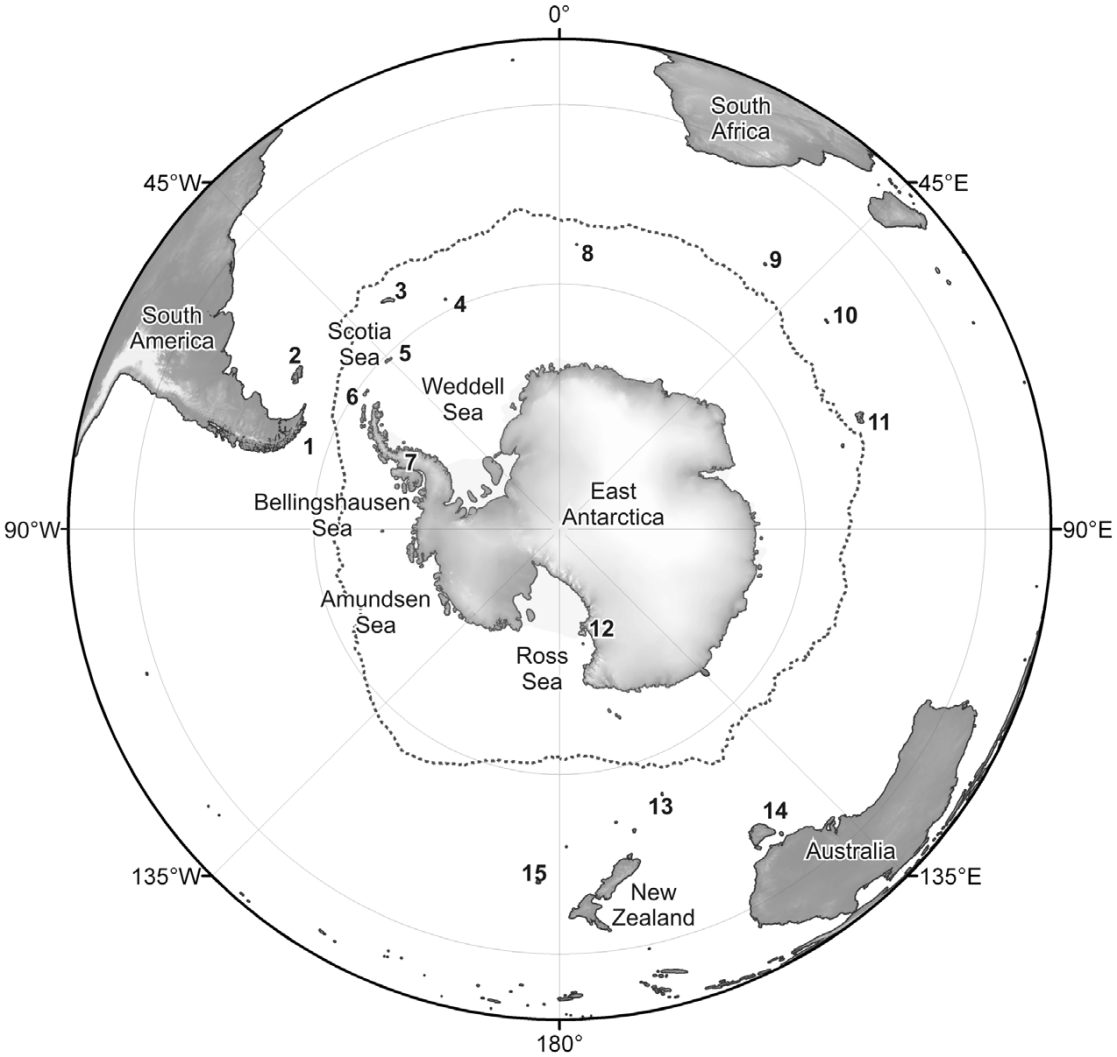
Map of the Southern Ocean and neighbouring regions. The dotted line around Antarctica represents the mean position of the PF. 1 = Tierra del Fuego, 2 = Falkland Islands, 3 = South Georgia, 4 = South Sandwich Islands, 5 = South Orkney Islands, 6 = South Shetland Islands, 7 = Antarctic Peninsula, 8 = Bouvet Island, 9 = Prince Edward Islands, 10 = Crozet Islands, 11 = Kerguelen Islands, 12 = McMurdo Sound, 13 = Macquarie Island, 14 = Tasmania, 15 = Chatham Islands.

**Figure 2:**
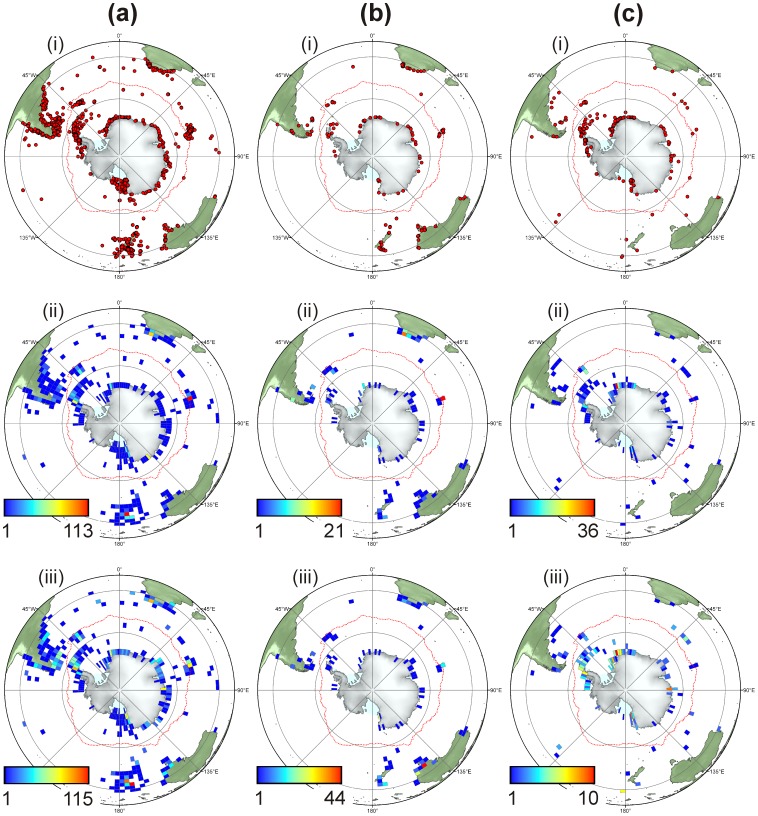
Quantifying the distribution of sponges within the Southern Ocean and neighbouring regions. Maps include three classes of Porifera (Demospongiae (ai–iii), Calcarea (bi–iii), and Hexactinellida (ci–iii)). (i) The distribution of sample locations for each class of sponge. (ii) The number of unique sample stations in a 3° by 3° grid cell for each class of sponge. (iii) The number of species collected from each 3° by 3° grid cell for each class of sponge.

In total 10,331 sponge records from the Southern Hemisphere were entered to the study database, of which 8,864 had been identified to species level (Table 1). This study included 8,864 data records for sponges from 349 research publications, expedition reports and online data sources (Figs. 1, 2). In total the database comprised geo-referenced records for 1570 sponge species (∼21% of global species) of which 397 were from the SO. Demospongiae dominated (70–75%) at all taxonomic levels from family to species within the SO.

**Table 1:** Summary of all the sponge records held in the database created for this study.

	Porifera	Demospongiae	Hexactinellida	Calcarea
Total records	10331	7331	1035	498
Antarctic Families	70	49	7	14
Antarctic Genera	139	99	21	19
Antarctic Endemic Genera	12 (8.6%)	5 (5.1%)	5 (24%)	2 (11%)
Antarctic species	397	296	50	51
Antarctic Endemic Species	170 (43%)	112 (38%)	34 (68%)	24 (47%)
Records not identified to class	1467 (14%)	–	–	–
Records not identified to Family	534	294 (4%)	212 (20%)	28 (5.6%)
Records not identified to Genus	117	88 (1.2%)	28 (2.7%)	1 (0.2%)
Records not identified to Species	596	464 (6.3%)	113 (11%)	19 (3.8%)
Total unidentified records	1467 (14%)	846 (12%)	353 (34%)	48 (9.6%)

The number of unidentified sponge records gathered in this study means that there is a large amount of data that could not be included in our analyses (Table 1). Within the part of the Southern Hemisphere considered here, over 10,000 records of sponge occurrences were recorded. Proportions of taxa not identified to species level varied between classes; Hexactinellida (34%), Demospongiae (12%) and Calcarea (10%).

Within this study, there are several families of SO sponges that are genera and/or species rich. Within the class Demospongiae representatives of 46 families were recognised and many of these were species rich, particularly the Chalinidae (31 species), Microcionidae (21 species), and Coelosphaeridae (21 species). Genera-rich families in the Demospongiae included the Polymastiidae (8 genera) and Hymedesmiidae (7 genera). However, within the 7 families of the class Hexactinellida, most families were genus- and species-poor, apart from the Rossellidae (10 genera, 40 species). The 14 class Calcarea families were neither genus- nor species-rich when compared to the Demospongiae. The most species-rich families were the Achramorphidae and Leucettidae (9 species each). Numbers of genera per family were smaller than the Hexactinellida, with a maximum of 2 genera recorded in 5 of the families of Calcarea.

Sampling intensity (Figs. 2 ii a–c) within each of the 3° by 3° grid cells was low for all sponge classes. Only a few areas within the PF were well sampled, such as the Antarctic Peninsula, South Georgia, and eastern Weddell Sea, compared with large areas such as the western Weddell Sea and Amundsen Sea having virtually no sampling points. Similarly, sampling intensity in neighbouring regions varied greatly, with peaks found around Tierra del Fuego, the Falkland Islands, Iles Kerguelen, Cape Town (South Africa) and North Island (New Zealand). However, all other areas were poorly sampled. The majority of sampling points were in coastal areas, with deep-sea sampling being far patchier.

Species counts (Figs. 2 iii a–c), per grid cell indicate that the highest numbers of species occurred in relatively localised areas. Demospongiae species counts largely reflected sampling intensity and were higher around the Antarctic Peninsula, Cape Town, Iles Kerguelen and North Island (NZ), with smaller hotspots found in the East Antarctic and the Ross Sea (Fig. 2 iii a). High species counts for Calcarea were found in SW Australia despite low numbers of sampling records from the region (Fig. 2 b iii). High species counts for Hexactinellida occurred around the Antarctic Peninsula, East Weddell Sea, East Antarctica, Ross Sea, and Kermadec Islands, and were mostly from cells that did not show particularly high levels of sampling.

**Figure 3:**
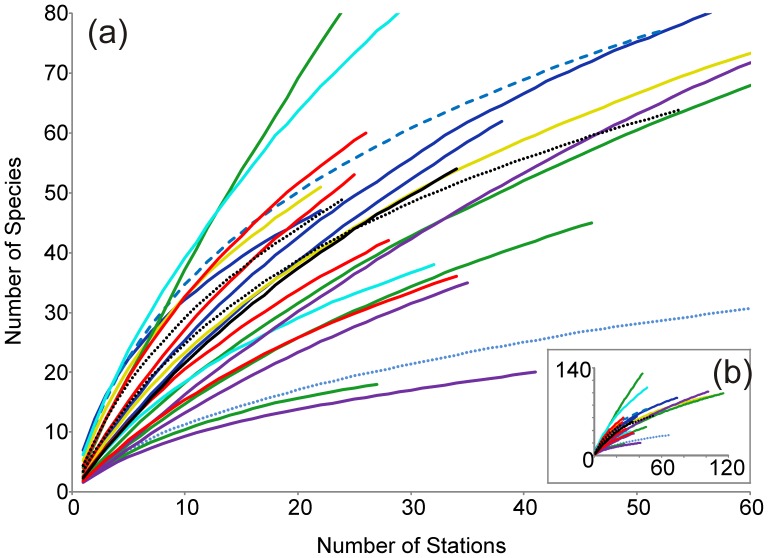
Rarefaction curves which show the accumulation of sponge species (all 3 classes of sponge were used) for selected 3° by 3° grid cells from the Southern Ocean and neighbouring regions. Rarefaction curves are coloured by region: Dark blue (solid) = Antarctic Peninsula; Blue (dashed) = East Weddell Sea; Blue (dotted) = East Antarctic; Turquoise (solid) = Ross Sea; Black (solid) = South Shetland Islands; Black (dotted) = South Georgia, Yellow (solid) = Kerguelen Islands; Red (solid) = South America and the Falkland Islands; Green (solid) = New Zealand (North Island); and Purple (solid) = South Africa.

Rarefaction analysis (Figs. 3a, b) indicated no obvious regional grouping of species richness trends. As most grid cells had less than 60 sampling sites, Figure 3a focuses on this section of the rarefaction curves, with Figure 3b depicting the full data extent. None of the curves obtained reached an asymptote, indicating that the sampling available to date is still not intensive enough to capture most species present within each grid cell. However, some generalised conclusions can be drawn: most Antarctic grid cells that have been sampled have high levels of species richness (Antarctic Peninsula, South Shetland Islands, South Georgia, East Weddell Sea and Iles Kerguelen), all between 37–50 species for 20 sampling stations. Antarctic grid cells sampled from within the Ross Sea illustrate that one region can have markedly different species richness trends. Here, one grid cell had the highest level of localised species richness found in the Antarctic, while the other grid cell gave amongst the lowest. The only grid cell sampled from East Antarctica had the lowest species richness found in the Antarctic region.

New Zealand grid cells (all located around North Island) had the widest ranges of species richness, and included the steepest rarefaction curve, indicating particularly high localised species richness. However, the remainder of well-sampled New Zealand grid cells typically displayed lower species richness than most Antarctic grid cells. South American grid cells (including the Falkland Islands) had a wide range of species richness. The highest was found within the Falkland Islands and Tierra del Fuego, comparable to that found within Antarctica. South African grid cells tended to have low levels of species richness compared to those found in most of Antarctica.

### Antarctic Endemism

Of the 397 Antarctic sponge species included within this study, 43% (170 species) were determined to be endemic to Antarctica (i.e. occurring solely within the PF) (Table 1). Differences in levels of endemism between the sponge classes are particularly striking, with 68% (34 species) of Hexactinellida endemic, followed by 47% (24 species) of Calcarea and 38% (112 species) of Demospongiae. Generic endemism was determined to be ∼9%, with hexactinellids having the highest proportion of generic endemism (24%), followed by Calcarea (11%) and demosponges (5%). Demosponge endemic genera included *Cladothenea, Acanthorhabdus, Pachypellina, Astrotylus* and *Raspailia (Hymeraphiopsis)*. Hexactinellid endemic genera included *Acoelocalyx, Docosaccus, Uncinatera,* and *Anoxycalyx (Scolymastra)* and *Rossella* s. str. (excluding *R. nodastrella*, which is non-monophyletic with the Antarctic *Rossella* spp.). Calcarea endemic genera included *Jenkina* and *Dermatreton*. All endemic hexactinellid and demosponge genera were monotypic, whereas those of Calcarea were polytypic.

### Species Range

**Figure 4:**
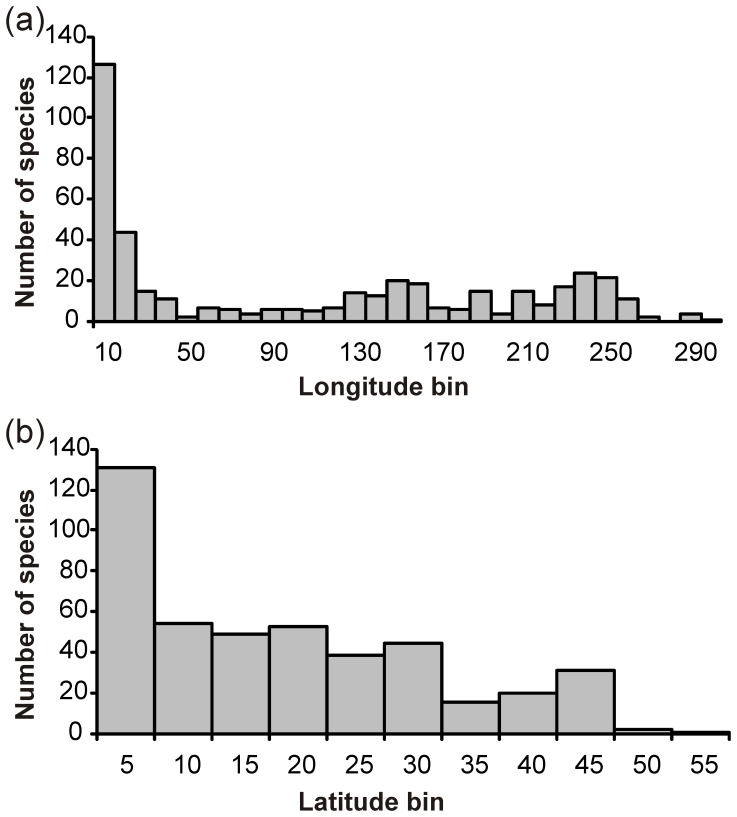
Latitudinal and longitudinal range sizes of sponge species found at three or more locations in the Southern Ocean and neighbouring regions. (a) Longitudinal range and (b) is the latitudinal range. Range size is the difference between the maximum and minimum range points and does not imply that the organism is found everywhere in-between.

The longitudinal distribution ranges of 441 analysed Southern Hemisphere species (those with 3 or more records) show that 125 species (28%) had limited ranges (<10°), including 40% of the Calcarea species, 28% of the Demospongiae and 15% of the Hexactinellida. The 104 species with wide longitudinal ranges (>200°) included all sponge classes evenly (Fig. 4a). Examples are found in Demospongiae (e.g. *Iophon radiatum*: 291°), Hexactinellida (e.g. *Rosella racovitzae*: 290°) and Calcarea (e.g. *Leucetta leptoraphis*: 229°). The latitudinal distribution ranges of sponge species illustrated that a number of species have wide ranges in the Southern Hemisphere. Overall, 34 species (8%) had latitudinal ranges >40°. However, there were a greater number of species (165 species or 37%) with limited latitudinal ranges (<10°). Within these Calcarea contained the largest proportion of species (64%), followed by demosponges (40%), then Hexactinellida (30%). Wide latitudinal ranges were found in all sponge classes (hexactinellids 15%, demosponges 8%, Calcarea 4%), with the widest ranges found in Demospongiae (e.g. *Suberites caminatus*: 51°). Examples for high ranges in Hexactinellida included *Anoxycalyx ijimai* (44°) and in Calcarea *Clathrina coriacea* with 46°.

**Figure 5:**
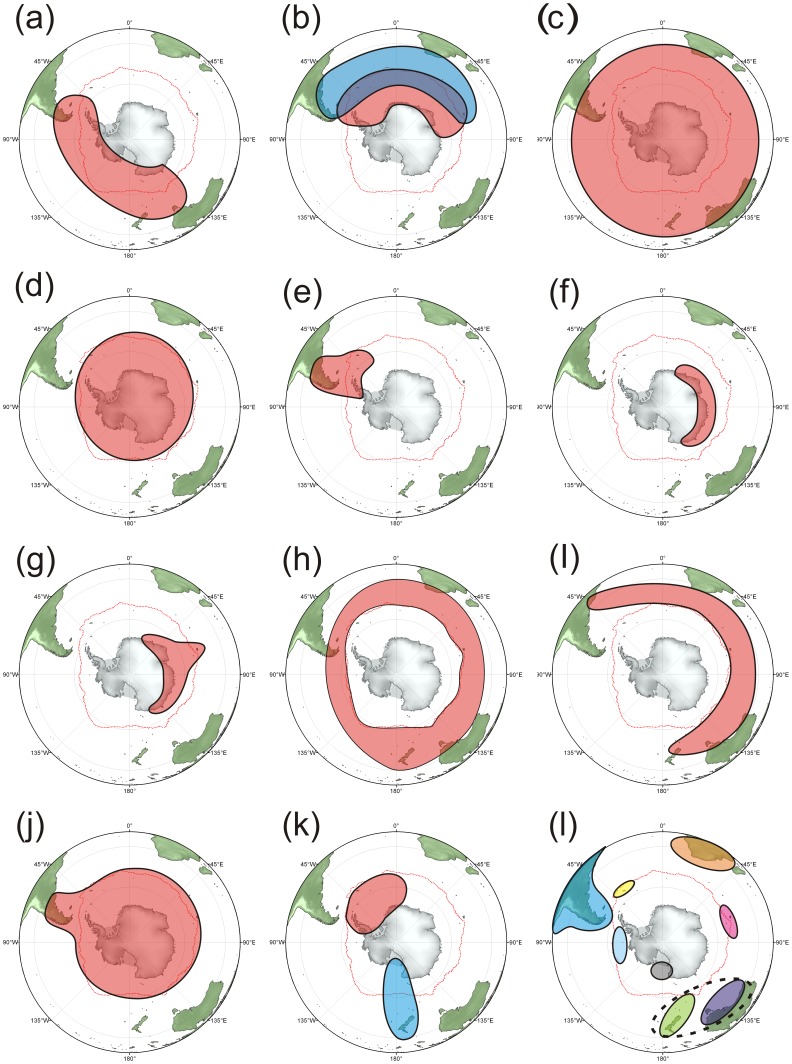
General distribution patterns of sponge species found at three or more locations within the Southern Ocean and neighbouring regions. The numbers of species per patterns are: a = 52, b = 53, c = 51, d = 47, e = 26, f = 11, g = 10, h = 6, i = 6, j = 4, k = 11 (5 blue, 6 red), l = 156 (27 South America, 2 Bellingshausen Sea, 8 Southern Australia, 11 Australia-New Zealand, 45 South Africa, 10 Kerguelen Islands, 51 New Zealand, 1 South Georgia, 1 Ross Sea).

We mapped the distribution of all species with 3 or more sample points to determine distribution patterns and similarities between taxa. Nineteen distinct species distribution patterns were found (Figs. 5a–l, Table 2). Nearly half of the distribution patterns were represented by less than 10 species, with the remainder based on 10–53 species. All but one of the patterns observed were dominated by demosponge species ranging from 50 to 100% of the species. The species rich genera of *Haliclona* and *Clathria* (Demospongiae) were major contributors to many of the patterns. Regional endemics were most common in regions to the north of the study, possibly due to an effect of the scope of the database and not true endemism. Four of the patterns (Fig. 5c, d, h & j) showed circumpolarity (108 species) reflecting the patterns shown in Fig. 4a.

**Table 2:** Summary of the major distribution patterns of sponge species found at three or more locations within the Southern Ocean and neighbouring regions (also see Fig. 5).

Pattern	Number of species	Dominant class	Dominant families	Dominant genera	Description
A	52	Demospongiae (85%)	Chalinidae, Niphatidae & Rossellidae	*Haliclona*	Tasmania eastwards to South America/Antarctic Peninsula
B	53	Demospongiae (94%)	Chalinidae & Microcionidae	*Clathria & Haliclona*	South America/Antarctic Peninsulaeastwards to Kerguelen Plateau
C	51	Demospongiae (90%)	Microcionidae & Suberitidae	*Clathria & Mycale*	Circumpolar: north and southof the Polar Front
D	47	Demospongiae (91%)	Coelosphaeridae & Isodictyidae	*Lissodendoryx*	Circumpolar: south of the Polar Front
E	26	Demospongiae (96%)	Halichondriidae & Microcionidae	*Clathria*	South America/Scotia Sea/AntarcticPeninsula
F	11	Demospongiae (73%)	–	–	East Antarctica: 20°–140°E
G	10	Demospongiae (80%)	Suberitidae & Chalinidae	*Haliclona*	East Antarctica/Kerguelen Plateau
H	6	Demospongiae (100%)	–	–	Circumpolar: north of the Polar Front
I	6	Demospongiae (66%)	–	–	South America eastwards to New Zealand:sub-Antarctic/north of Polar Front
J	4	Demospongiae (75%)	–	–	Circumpolar: south of the Polar Frontand South America
K	Weddell Sea/Antarctic Peninsula: 6	Demospongiae (50%)	Ancorinidae & Isodictyidae	–	Weddell Sea/Antarctic Peninsula
	Ross Sea/New Zealand: 5	Demospongiae (60%)	Polymastiidae	–	Ross Sea/New Zealand
L	Total: 156	Demospongiae (78%)	Ancorinidae	*Sycon & Isodictya*	Regional endemics
	Australia: 8	Demospongiae (63%)	Microcionidae	–	Regional endemics
	Australia & New Zealand: 11	Demospongiae (55%)	Pleromidae & Heteropiidae	*Grantessa & Pleroma*	Regional endemics
	New Zealand: 51	Demospongiae (96%)	Suberitidae	*Polymastia, Psammocinia* *& Aaptos*	Regional endemics
	South Africa: 45	Demospongiae (73%)	Ancorinidae, Irciniidae, Phymatellidae & Darwinellidae	*Sycon & Isodictya*	Regional endemics
	South America: 27	Demospongiae (89%)	Chalinidae & Mycalidae	*Mycale & Haliclona*	Regional endemics
	South Georgia: 1	Demospongiae (100%)	Microcionidae	*Clathria*	Regional endemics
	Bellingshausen Sea: 2	Hexactinellida (100%)	Isodictyidae & Uncinateridae	*Uncinatera & Pararete*	Regional endemics
	Kerguelen: 10	Demospongiae (70%)	Chalinidae	*Haliclona*	Regional endemics

**Figure 6:**
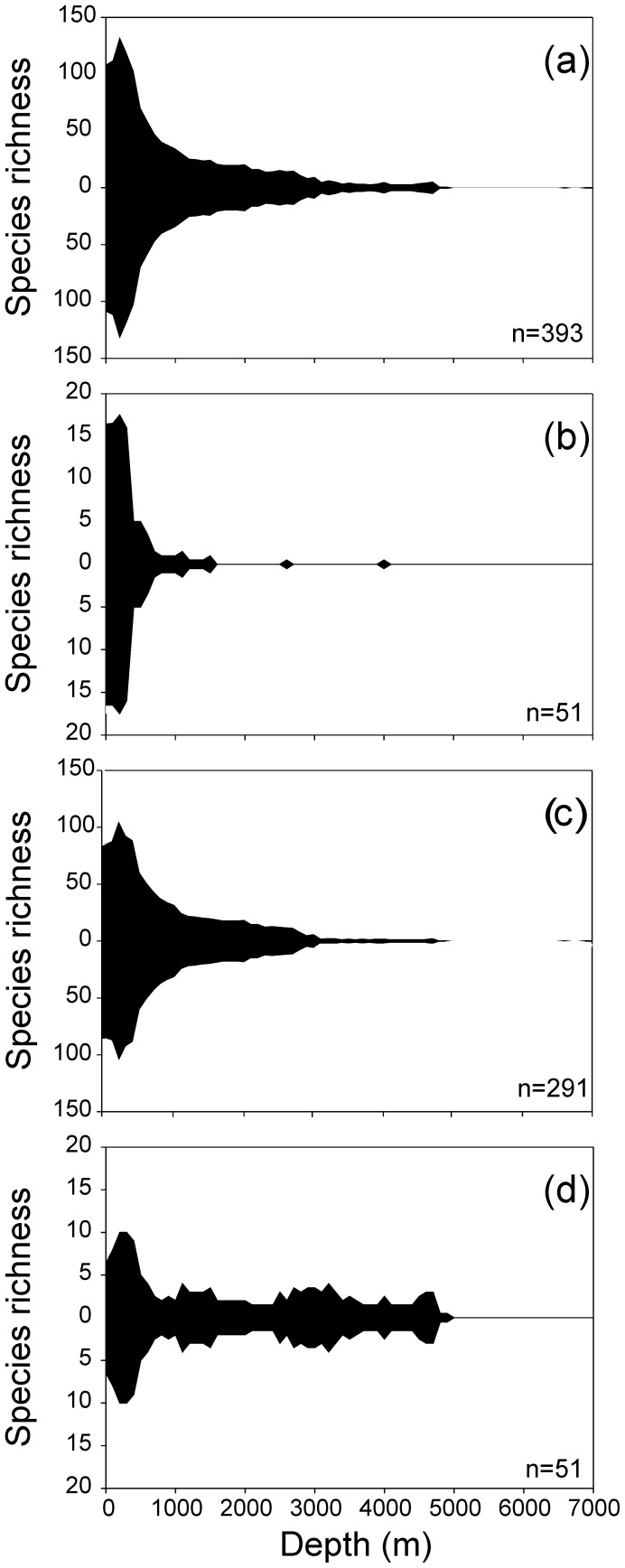
Depth distributions of sponges in the Southern Ocean. All Porifera (a), and each class of Porifera: Calcarea (b), Demospongiae (c), and Hexactinellida (d).

**Figure 7:**
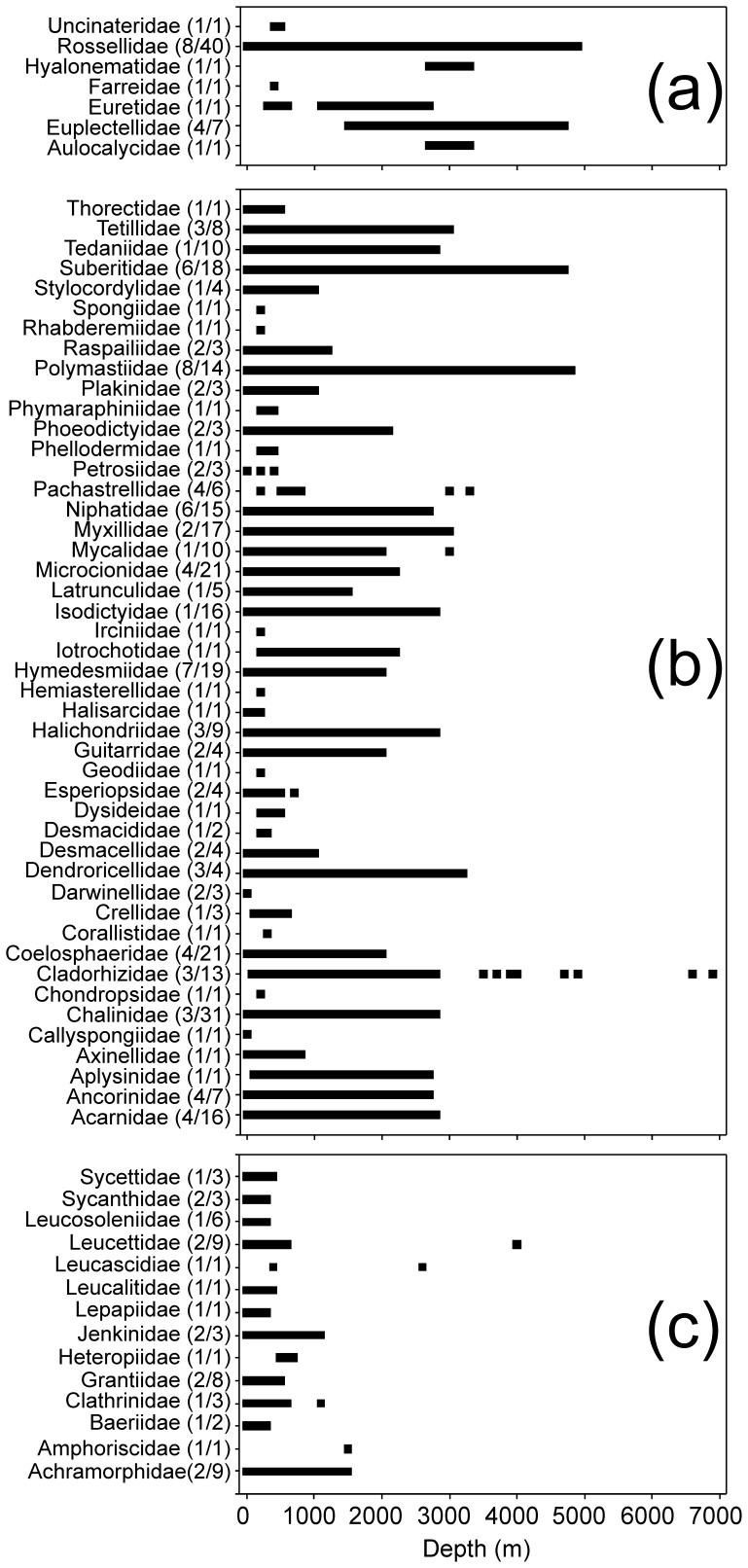
Depth ranges of each sponge family within their respective class. Hexactinellida (a), Demospongiae (b), and Calcarea (c) in the Southern Ocean. Brackets after family name indicate firstly the number of genera within that family, and secondly, number of species within that family.

In order to explore the nature of eurybathy in SO sponges, we analysed maximum and minimum depth as well as the depth range at which each species was found (Figs. 6a–d, 7a–c), as well as defining their presence on the shelf, slope and abyss in order to determine eurybathy. Thirty-six percent of all recorded sponge species were found to have small ranges (0–100 m). Twenty-nine percent of all species had depth ranges over 500 m, with only 15% having depth ranges that were greater than 1000 m. Eight percent of all sponges were found to be shallow species, having a maximum recorded depth that is less than 100 m. Sixty-four percent of species were restricted to the shelf, and 25% of species were found to be distributed in more than one zone (shelf, slope or abyss), with the majority of these found at both shelf and continental slope locations.

There were notable differences in depth distributions between classes, with particular families driving these major trends. Only 4% of calcareous species had depth ranges that are greater than 1000 m, and 10% had depth ranges that are over 500 m, driven by the families Achramorphidae, Jenkinidae, Leucettidae and Grantiidae families (Fig. 7b). A much larger group of calcareous sponges (33%) had very limited depth ranges of less than 100 m, which indicates limited eurybathy. Eighty-eight percent of Calcarea species were restricted to the shelf, and 6% were only found shallower than 100 m, with only 6% found in more than one bathymetric zone.

Fourteen percent of hexactinellid species had depth ranges greater than 1000 m, and 24% had depth ranges greater than 500 m. These wide depth trends were driven by the families Rossellidae, Hyalonematidae, Euretidae, Euplectellidae, and Aulocalycidae. However, 45% of Hexactinellida species had depth ranges that are less than 100 m. Forty-two percent of Hexactinellida had maximum depth records of less than 1000 m (over a third of these were restricted to the shelf), which indicates that Hexactinellida are more likely to be found at greater depths than the Calcarea. Over a quarter of representatives of this class were restricted to the abyssal plains and 28% of Hexactinellida species were distributed in more than one depth zone.

Eighteen percent of demosponges had depth ranges greater than 1000 m, and 19% had depth ranges greater than 500 m, which indicates that representatives of this class showed the highest occurrence of eurybathy. This trend for Antarctic eurybathy was driven by over 20 families of Demospongiae, particularly by the Suberitidae, Polymastiidae, Tedaniidae, and Coelosphaeridae. This class also has the highest depth range recorded for a single species of ∼4900 m (*Polymastia invaginata*), compared to 4300 m (*Bathydorus spinosus*) in Hexactinellida and 1500 m (*Achramorpha truncate*) in Calcarea. Seventy-three percent of Demospongiae sponges had maximum depths of less than 1000 m, and 9% had maximum depths that are less than 100 m. The deepest (>6000 m) recorded sponge species (*Asbestopluma wolffi* and *A. hadalis*) were both found within the demosponge family Cladorhizidae. Sixty-four percent of all demosponge species were restricted to the shelf, with 25% of species distributed in more than one depth zone.

### Southern Ocean Sponge Biogeography

**Figure 8:**
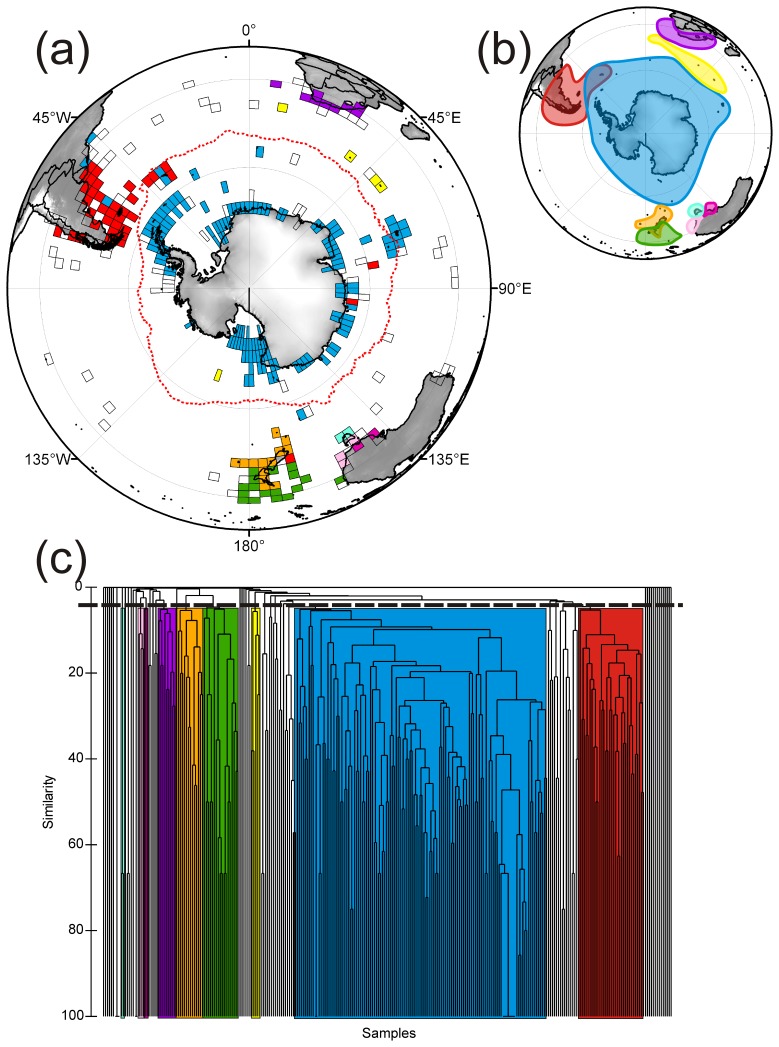
Large scale biogeographic relationships in demosponge species assemblage in 3° by 3° grid cells from the Southern Ocean and neighbouring regions. Each grid cell contains 3 or more species of demosponge. (a) Geographic representation of the relationships shown in c. (b) A simplified representation of the biogeographic relationships found in a. (c) Cluster analysis of the percentage faunal similarity between grid cells. The colours represent each geographic region: Antarctica (blue), South America (red), South Africa (purple), sub-Antarctic (yellow), New Zealand temperate (orange), New Zealand tropical (green), Southern Australia (dark pink), Tasmania (light blue), and SE Australia (light pink).

In order to discern biogeographic patterns within the sponges, PRIMER was used to analyse the species composition of the 3° by 3° grid cells (Figs. 8 a–c). Only the results for the Demospongiae are presented here as limited numbers of species level records for the Calcarea and Hexactinellida meant that biogeographic patterns could not be ascertained.

Nine distinct geographic groupings of demosponges (Fig. 8a) were evident using a cut-off point of ∼4% similarity (Fig. 8c) from the 303 grid cells that were suitable for the analysis. Group 1 (blue) is the largest biogeographic group, and encompasses the area found within the PF (Antarctic Group), but extends beyond the continental shelf to include Iles Kerguelen and Macquarie Island, as well as grid cells from sub-Antarctic South Georgia (south of the PF) (Fig. 8b). Biogeographic group 2 (yellow) contains a small number of Sub-Antarctic island grid cells (Prince Edward Islands and Iles Crozet) found between South Africa and Antarctica. Group 3 (red) includes South America, the Falkland Islands, and 4 cells from around South Georgia. Groups 4 (orange) and 5 (green) comprise similar sized groups that overlap between North and South Islands of New Zealand. Group 6 (dark pink) consists of 2 grid cells of South Australia. Group 7 (light pink) consists of 3 cells from SW Australia. Group 8 (light blue) consists of cells around Tasmania. Group 9 (purple) consists of several grid cells located at the tip of South Africa.

## Discussion

### Data, Taxonomy, Sampling Coverage

This study represents the most comprehensive database and analysis of Southern Hemisphere sponges attempted to date. Collating sponge distribution data presented several challenges. The available data were often poor quality with a low level of taxonomic resolution and/or a low spatial resolution. As a result sufficient data was only available to analyse Demospongiae biogeographic patterns. As has been observed more generally in SO studies, the distribution of sponge sampling in the Southern Hemisphere is patchy and uneven, with a paucity of data available from the deep ocean [35]–[37]. Our analyses identified that certain regions in the SO remain under-sampled for sponges, particularly the Amundsen Sea, parts of East Antarctica, and almost all deep-sea areas. Even relatively well-sampled areas have not yet been sampled sufficiently to be confident that the majority of species have been found (Fig. 3).

### Species Richness and Endemism

Several studies have assessed the richness of Antarctic sponges, giving ranges from ∼250–530 species [2], [3], [12], [17], [32], [38]. In this study, we identified records of 397 distinct Antarctic species, representing 139 genera in 70 families. This differs from previous estimates due to several factors, including different definitions of the boundaries of Antarctica [32], increased sampling [36] and improved taxonomic knowledge (www.marinespecies.org/porifera/). Increases in knowledge are most dramatic within the classes of Hexactinellida and Calcarea, and have doubled and even tripled the number of species found in the SO in recent years [18], [19], [39].

Clarke and Johnston [17] calculated that 2.6–5.6% of total world sponge species are found in the SO (based on an estimate of 250 Antarctic species, which excluded Hexactinellida, and on an estimate of 5–10,000 Porifera species worldwide). New estimates from the current study (including Hexactinellida) suggest that 5.2% of all sponge species are found within the SO (www.marinespecies.org/porifera/accessed October 2011), which is low when considering that ∼9% of global continental shelf area is found in the Antarctic (Barnes and Peck 2008). SO Hexactinellida (8.4%) and Calcarea (7.6%) proportions of world species are in line with the amount of SO continental shelf available; however, the class Demospongiae (3.9%) has a lower proportion of species than expected. This low proportion of SO species is probably due to the enormously high demosponge diversity in the tropics, especially reef environments, but compared with the Arctic or Atlantic boreal sponge faunas, the Antarctic sponge diversity is actually high. This low level shelf species richness is not only seen in sponges, but is a feature of many SO benthic fauna, such as bivalves, ascidians, and ophiuroids [17], [40]. However, fauna such as pycnogonids (20%), polycheates (12%), and bryozoans (∼10%) have values that are greater than what would be expected on the shelf [41].

There was no obvious regional pattern in sponge species richness, unlike that found in, for example, Pycnogonida in the Southern Hemisphere [42]. However, none of the rarefaction curves calculated here approaches its asymptote. Species richness in the Pycnogonida is higher in Antarctica compared to other regions in the Sothern Hemisphere and, within this, the eastern Weddell Sea and the South Orkney Islands were seen as the richest regions. In contrast, Antarctic sponge species richness is similar to other Southern Hemisphere regions, with no evidence of a distinct cline in species richness with increasing latitude. A wide spectrum of species richness was found within sub-regions of Antarctica and neighbouring regions, possibly associated with known distributions and biomass patchiness (e.g. [4]), which are driven by ecological processes.

Variability in large scale species richness and diversity depends on depth as well as latitude [43]. Previous studies have indicated that there is likely to be a depth cline in sponge diversity, with abyssal depths exhibiting lower levels of diversity [12], [44]. Unlike SO demosponge and calcareous sponges, which are more restricted to the shelf, hexactinellids are found to be spread more evenly across depths, and data indicate an increase their higher level taxonomic diversity with depth, specifically from slope to abyss [3], [5]. A greater number of hexactinellid families are found at slope depths than at abyssal depths, but the ratio of genera to families at abyssal depths (3∶1) is greater than that found on the slope (2∶1). More hexactinellid species are found at abyssal depths than on the slope, however, the greatest numbers of species, and the greatest ratio of species to genera (3∶1) are still found at shelf depths. No diversity clines with depth have been reported in other SO fauna, such as bivalves [45], and isopods [12]. Reasons suggested for the higher diversity with depth in hexactinellids than in other sponges have included increased area availability at abyssal depths [36], deep-dwelling characteristics in hexactinellids [16], silica availability in the SO [46], and higher food availability in some of their most abundant regions, the Weddell and Ross Seas, due to deep-water production [12].

Previous studies have indicated that the Antarctic marine fauna typically includes relatively high numbers of endemic species (50–80%) but with a considerably lower level of generic endemism [17], [38], [47]. However, more recent studies have suggested that species endemism levels are more likely to be closer to 50% [41]. Our estimate of species-level endemism was lower (43%) in sponges within the PF, which included shelf, slope and abyssal species. This is similar to levels found in cyclostome bryozoa, bivalvia [41], and ascidians [48]. Our level of sponge generic endemism (∼9%) is lower than some SO fauna, such as amphipods (17%) [49] and molluscs (13%) [50], but is the similar to cheilostome Bryozoa (8%) [51]. Local endemics were found within the Iles Kerguelen (10 species), Bellingshausen Sea (2 species), Ross sea (1 species), and South Georgia (1 species) (Fig. 5l). The PF has been cited as an important barrier to species migration in and out of the SO (e.g. [36], [52]), as there is a sharp temperature gradient across it [53], which decreases with depth [54]. Large numbers of sponges within this study were found to have PF-dominated distributions, and fairly restricted latitudinal and bathymetric ranges. Many are also thought to be stenothermal species [10], suggesting that the PF and deep water surrounding Antarctica form an effective barrier to many species.

### Species Ranges

The ACC is linked with other currents and coastal gyres (e.g. the Antarctic Coastal Counter Current), giving species the potential to be widely distributed longitudinally along the entire Antarctic coastline [41]. Within the PF, relatively homogenous conditions (temperature, salinity, nutrients) mean that marine organisms have the potential, given enough time and dispersal capability, to occur within the whole of the SO. Therefore, the dominance of range-restricted species and the small number of species found to have wide ranges in the current study is surprising. However, this pattern is not unique, and has been found in other SO fauna, such as gastropods, bivalves [55] and pycnogonids [42]. Sponges have more restricted longitudinal ranges in the SO compared to pycnogonids and bivalves, but are similar to those found in gastropods. However, at least a quarter of sponge species have very wide longitudinal ranges and, with the notable clustering of species that probably have circumpolar distributions (>200°) (Fig. 4a), similar patterns are observed in pycnogonids, bivalves and gastropods. Latitudinal ranges in sponges are also relatively restricted (Fig. 4b), again similar to that found in gastropods. Bivalves and pycnogonids have a different range structure, and both include a greater number of latitudinally wide-ranging species. Despite the indication that fewer sponges and other benthic fauna may have circumpolar distributions than previously thought, it is believed that with increased sampling coverage, particularly in the Amundsen and Bellingshausen Seas, more species will be included in this category [55].

Dispersal mechanisms are an important biological parameter in understanding range and distribution patterns. These mechanisms are varied, ranging from lecithotrophic larvae from both oviparous and viviparous sponges [56]–[58] and asexual reproduction through bipartition and budding [10], [30], [59]–[61]. Lecithotrophic larvae generally have a short-lived existence, which could be important in understanding why some species are range restricted. However, little is known about the longevity of this phase in Antarctic sponge larval duration, which could potentially be far longer than is currently known for temperate species and therefore could be important in creating and maintaining circumpolar distributions [2], [62]. Recent investigations of hexactinellid sponges in the Weddell Sea indicate the possible important role of asexual reproduction in this region [61]. Flotation of these negatively buoyant sponge buds, through macroalgal rafting, has been noted within the sub-Antarctic which, coupled with potentially rapid colonisation rates, could explain the wide ranges of certain sponge species [2], [63]. However, these apparently wide-ranging species could also include examples of cryptic speciation [32]. There has so far been study on cryptic speciation in Antarctic sponges, but those undertaken in other regions indicate that cryptic species do exist (e.g. [64], [65]) and are potentially more common than previously considered [66].

The vast majority of Antarctic sponge species distributions are consistent with the impacts of west wind drift and the ACC (Figs. 5a–d, h–j). The less prevalent SO-dominated distributions (Figs. 5f, g, and k (red)), are also underlain by the ACC, which generates large gyres, driving the Antarctic Coastal Counter-Current. The importance of the Kerguelen Plateau as a regionally shallow area for sponges to colonise in the SO is indicated in Fig. 5k (red). Fewer distributions are localised (Fig. 5l), and indicate regional endemism. Islands such as Kerguelen and South Georgia may be able to develop endemics due to their relative size and isolation within the SO. Fig. 5k (blue) is intriguing, as there are no obvious oceanographic explanations for this pattern, although it has been previously observed in cheilostome bryozoans [47]. The possibility of creep along the Macquarie Ridge could explain this distribution pattern, and the small number of species that display this particular distribution also indicates that it is not a major dispersal pathway. Distribution A (Fig. 5a), also suggests possible linkages between Antarctica and New Zealand and Southern Australia, which has not been observed in other taxa [41]. A Scotia Sea centred pattern (Fig. 5e) reflects, as noted by Koltun [10], that Antarctic sponges have their strongest relationship with the Falklands and South America. Other distribution patterns also show Antarctica as having a strong relationship with the southern tip of South America. The explanation for such a strong relationship with South America is likely to be a combination of South America being the last continent to break away from Antarctica, and the potential existing for species to cross the PF by ‘island hopping’ along the Scotia arc [47], [50].

Eurybathy has been thought to be a particularly important characteristic within SO sponge ranges [2], [10], [32], [67]. For the first time, our analyses demonstrate that eurybathy is an important but not a dominant characteristic of SO sponges. The prevalence of eurybathy differs strongly between sponge classes (Fig. 8), and certain families and classes have either strong eurybathic or stenobathic characteristics (Fig. 7). Oceanographic and sedimentological conditions in the SO are seen as important in promoting eurybathy in benthic fauna. Koltun [10] suggested the oceanographic reasons for sponge eurybathy are two-fold. Firstly, he believed that due to the absence of continental runoff, ocean waters extend all the way to the edge of Antarctica, which brings sponges from bathyal depths to shallower parts of the shelf. Second, the presence of strong bottom currents promotes the movement of benthic species from the shallows to the continental shelf [10]. Relatively uniform physical and chemical conditions on the shelf, and varied bottom sediments as substrata, promote eurybathy by reducing vertical zonality. This can be seen in certain families, which are found to be strongly eurybathic (range >5000 m), such as the Rossellidae, Suberitidae, Polymastiidae, and Cladorhizidae, and these are commonly found in other oceans at great depths [68]. Eurybathy is also seen as a possible evolutionary by-product of glacial-interglacial cycles of ice sheet advance and retreat, which eliminated most shelf fauna during glacial cycles, thereby periodically forcing species into deeper water to escape extinction [12], [67], [69]. Despite these processes driving eurybathy in SO sponges, many species are still found to not exhibit these characteristics.

Eurybathy is not a characteristic of all sponges, particularly the calcareous sponges. Calcarea tend to be strongly stenobathic, found predominantly on the shelf, and are the most latitudinally and longitudinally restricted of all the classes. These characteristics may have been important in driving the high levels of both species- and genus-level endemism in this class. Calcarea may also be restricted by the shallow CCD (calcium compensation depth) in the SO [36], [70], which could explain their predominance on the shelf (Figs. 6b, 7c). Globally, hexactinellid sponges are generally thought to be stenobathic and less likely to be found at abyssal depths [16]. However, this study suggests that SO hexactinellids have eurybathic tendencies, and are as eurybathic as SO demosponges. Our data indicate that hexactinellid species are not rare in the abyssal depths (3000–6000 m), with 55% of hexactinellids found at these depths in the SO.

Within this study, demosponges have been found to have some of the highest levels of eurybathy, and this group includes species with some of the widest longitudinal and latitudinal ranges. These factors may be important in driving the relatively low richness of demosponges within the SO, and in reducing endemism. Similarly, hexactinellids are also found to be eurybathic, with extended latitudinal and longitudinal ranges. Large latitudinal and longitudinal ranges could be explained by their abyssal tendencies and the use of asexual budding in dispersal. Despite similar characteristics found between these two classes, it is clear that different processes must be driving their diversity and distribution patterns. In sharp contrast, Calcareous sponges show the most limited distributions of all classes.

### Biogeographic Patterns Explained

Antarctic sponges were originally thought to comprise a circumpolar fauna with a high percentage of endemic species, but also with links to the deep sea, sub-Antarctic regions (e.g. Kerguelen), and the South American fauna [2], [10], [16], [32]. From the results of our analyses of the distribution of demosponges (the only group for which sufficient data are available) we propose a distinct Antarctic bio-region (Figs. 8a–c). Our analyses do not support the biogeographic sub-regions in Antarctica previously proposed by Koltun [10].

Kerguelen and Macquarie Island, which have in earlier studies been classed as a separate sub-Antarctic region, which also included the Prince Edward Islands and Crozet (e.g. [71]), were found to be strongly Antarctic in faunal composition. A strong Antarctic influence has been reported in other taxa on Iles Kerguelen, such as bivalve molluscs and cyclostome bryozoans, and has been explained by the relative proximity of the Kerguelen Plateau to continental Antarctica (which has changed little through time); and a shallower seafloor between Kerguelen and Antarctica in the past [41]. This proximity has allowed the colonisation of sponges between Kerguelen and East Antarctica along the Kerguelen Plateau. Macquarie Island has also found to be faunisitically similar to Antarctica in other taxa, such as cheilostome bryozoa [47], due to colonisation along the Macquarie Ridge. The Prince Edward Islands and Iles Crozet form as a distinct bio-region, despite their relative proximity to Iles Kerguelen. The New Zealand sub-Antarctic islands are classified as part of the New Zealand temperate sponge fauna, rather than being included in the sub-Antarctic region (cf. [71]).

Previous sponge studies have combined the Antarctic and Magellan regions into a single province [32]. This study confirms the importance of South American influence within the PF, particularly at South Georgia (Fig. 8a–c). This has been illustrated by a recent study highlighting the importance of South Georgia’s biogeographic position within the PF, particularly in understanding both the southern range limits of the South American fauna and northern ranges limits of Antarctic species [72]. South Georgia is seen in this study, as a mixing ground for both South American and Antarctic sponge communities [73]. The importance of South American sponge fauna within the PF has been noted previously (e.g. [10]), and has also been reported in a range of other Antarctic fauna (e.g. [17], [41], [50], [74]). It has been hypothesised that fauna have migrated along the Scotia arc ‘stepping stones’ from South America into Antarctica [73], [75]. Like many other studies, our analyses found little faunal similarity between Antarctica and other Southern Hemisphere regions, including South Africa, New Zealand, and southern Australia (cf. [41], [76]). This largely reflects the timing of past continental connectivity in the Southern Hemisphere.

### Future Considerations

The use of molecular techniques in marine biology has clearly shown that the perceived single ocean with few boundaries to limit mixing within and between species does not exist [65]. Molecular studies on Antarctic invertebrates have indicated that many of the species currently known with wide ranges represent species complexes [77]–[79]. Sponge species complexes of previously widely distributed species have been found outside the SO (e.g. [80]), however, there is no published research yet on SO sponges. Range and depth restricted species were found to be more common in this study, than the previously believed [2]. Currently there are still many SO sponges which have either wide longitudinal and/or latitudinal ranges, and are eurybathic, which require the application of molecular techniques to determine if they are one continuous species or several different species. Over 7,600 marine sponges are identified as valid species [81], with recent studies indicating that sponge diversity could be double that currently known (e.g. [82]). In the near future, the number of Antarctic species, genera, families, and endemics are likely to increase, with the combined efforts of both morphological and molecular techniques (e.g. http://www.spongebarcoding.org/). Future work in these key areas could alter and improve our current understandings of SO sponge diversity, distribution and biogeography.

### Conclusions

Our analyses support the recognition of a single Antarctic demosponge biogeographic zone, which also includes the sub-Antarctic regions of Iles Kerguelen and Macquarie Island. The wider biogeographic divisions recognised are otherwise similar to previous studies on demosponges. Our data indicate that the biogeographic sub-Antarctic region comprised of the Prince Edward Islands and Iles Crozet to be distinct from the Antarctic and other sub-Antarctic islands. The New Zealand sub-Antarctic islands form a southern extension of the temperate New Zealand demosponge biogeographic region. South Georgia and Shag Rocks are presented for the first time as a region of overlap between the South American and Antarctic biogeographic regions in SO sponges. Our data are consistent with previous research in sponges and many other benthic groups indicating a strong faunistic link between Antarctica and South America. Levels of endemism indicate that the PF is an effective barrier to sponges colonising to and from other Southern Hemisphere regions. However, strong faunal connections between Antarctica and Iles Kerguelen and Macquarie Island, which currently lie north of the PF, indicate past and possibly ongoing connections. The position of the PF is variable over a range of timescales and is known to have been at least four degrees further north in the past [83].

Contrary to widely held perceptions, the majority of SO sponges have limited distribution and depth ranges, and eurybathy and circumpolarity are not general characteristics as previously thought. Forty-three percent of sponges are endemic to the SO, with hexactinellids having the highest species-level endemicity (68%). There are distinct depth range differences between sponge classes, with demosponges being more likely to be eurybathic than hexactinellids or Calcarea. Shallow maximum depths are recorded for many demosponges and calcareous sponges.

## Materials and Methods

### Data Acquisition

All publically available sponge occurrence records in the SO and adjacent oceans south of 25°S were compiled into a Microsoft Access database by the researchers of this study. These data were collated using published records and publically available expedition reports, with all nominal records having had their taxonomic status verified against the world register of marine species (www.marinespecies.org). During the timescale on which this work was carried out the taxonomic editors of the register have begun to make significant revisions of sponge taxonomy. The data used in this study were nomenclaturally correct as of June 2011 according to the World Porifera Database (www.marinespecies.org/porifera/). Sponge occurrence data were compiled using records that spanned over 130 years, from the earliest records from the Challenger expedition (1881), to the present day (including all taxonomic revisions to the original specimens). The authors of this study have made all data used within this study freely available through the open access SCAR-MarBIN website (scientific committee on Antarctic research marine biodiversity information network (www.scarmarbin.be)).

Over 8,864 sponge records, from sampling locations around Antarctica, the sub-Antarctic Islands, South America, South Africa, New Zealand and southern Australia were used within this study (Figs. 1, 2). The criteria applied to sponge records to be included in this study were (a) identification to species level (using morphological methods), and (b) a geo-referenced (latitudinal and longitudinal) collection location. Sampling depth (where given) was used to determine the extent of eurybathy. Abundance data were not used in this study because the majority of records did not include abundance, or did not use comparable sampling methods or means of recording abundance. The class Homoscleromorpha is recorded only by one genus with only sporadic Antarctic records, it is therefore excluded from all following analyses.

### Species Richness and Sampling Intensity

In order to determine species richness and quantify sampling intensity, the SO was divided into grid cells of 3° latitude by 3° longitude [55]. Grid cells that mainly fall within the Polar Front (PF) are hereafter referred to as Antarctic, and together with those of sub-Antarctic islands are referred to as SO [35], [41]. Each sampling station is regarded as a distinct point of longitude and latitude, reducing possible issues with duplication from different data sources, but potentially leading to underestimation of sampling effort as multiple sampling techniques were used at some locations. Each species was counted only once within each grid cell in order to determine the distinct number of species. Due to sampling intensity varying dramatically in the SO and neighbouring oceanic regions, the rarefaction curve technique can be used as it allows comparison between cells with different numbers of samples [84]. In order to compare species richness between cells, rarefaction analysis with 999 iterations of the best-sampled grid cells was carried out in PRIMER [85]. In total, 23 of the best-sampled grid cells (>20 stations) were used in the rarefaction analysis. Most regions were represented by these 23 grid cells, however, due to low numbers of records in Southern Australia and Southern New Zealand; these regions were not included in this analysis.

Endemism rates for the Antarctic were estimated as the number of species within this database that were only found south of the PF.

### Species Depth and Geographic Range

In order to analyse patterns of depth distribution in sponges in the SO (both Antarctic and sub-Antarctic regions), the bathymetry was divided into 100 m intervals from 0 to 7000 m. Sponge data were divided into the three different classes: Calcarea, Demospongiae and Hexactinellida, and only species with 3 or more distribution and depth records were used. The number of species and families found in each depth interval was calculated. When breaks occurred in the depth distributions of two or more species within a family, this was recorded in the results. In order to further analyse the nature of eurybathy of SO sponges, a second technique was utilised [55], which categorised each species into three depth zones: shelf (<1000 m), continental slope (>1000 m and <3000 m), and abyssal (>3000 m). Sponges that were found to have depth distributions than encompassed more than one zone were also enumerated.

Longitudinal and latitudinal ranges were calculated for all sponge species that had been recorded at 3 or more stations. Latitudinal ranges were calculated by subtraction of the lowest latitudinal value (most southerly) from the highest latitudinal value (most northerly). As the SO covers a full 360° of longitude, calculations had to be constructed so that they did not determine taxa with a limited range (e.g. ∼179°E to ∼179°W) to be circumpolar. We therefore calculated the minimum continuous arc that included all the distribution points of the taxon [55]. This is done by calculating all possible longitudinal distances between neighbouring records to obtain the maximum longitudinal distance between any two of the records. This value is then subtracted from 360° to provide the actual longitudinal range. However, this method in calculating longitudinal range of species also prevents any species from having a truly circumpolar distribution, due to gaps in sampling (see Fig. 2).

The distributions of species with 3 or more records were mapped using a Geographic Information System (GIS). Species with similar geographic distributions were grouped into major faunal patterns.

### Sponge Biogeography

PRIMER 6 software [85] was used to analyse the geographic relationships amongst sponge species in the Southern Hemisphere. Faunal similarity between 3° by 3° cells was quantitatively measured using Bray-Curtis similarities of nom-transformed presence/absence data [86].
